# Establishment and validation of a predictive model for tracheotomy in critically ill patients and analysis of the impact of different tracheotomy timing on patient prognosis

**DOI:** 10.1186/s12871-024-02558-x

**Published:** 2024-05-17

**Authors:** Xing-Hua Chen, Jing-Jing Zhao, Cheng Chen, Li Yao

**Affiliations:** Department of Intensive Care Unit, Hefei Hospital Affiliated to Anhui Medical University, The Second People’s Hospital of Hefei, Hefei, Anhui 230011 China

**Keywords:** Intensive care unit, Predictive model, Invasive mechanical ventilation, Tracheotomy, Timing of tracheotomy, MIMIC-IV database

## Abstract

**Background:**

In critically ill patients receiving invasive mechanical ventilation (IMV), it is unable to determine early which patients require tracheotomy and whether early tracheotomy is beneficial.

**Methods:**

Clinical data of patients who were first admitted to the ICU and underwent invasive ventilation for more than 24 h in the Medical Information Marketplace in Intensive Care (MIMIC)-IV database were retrospectively collected. Patients were categorized into successful extubation and tracheotomy groups according to whether they were subsequently successfully extubated or underwent tracheotomy. The patients were randomly divided into model training set and validation set in a ratio of 7:3. Constructing predictive models and evaluating and validating the models. The tracheotomized patients were divided into the early tracheotomy group (< = 7 days) and the late tracheotomy group (> 7 days), and the prognosis of the two groups was analyzed.

**Results:**

A total of 7 key variables were screened: Glasgow coma scale (GCS) score, pneumonia, traumatic intracerebral hemorrhage, hemorrhagic stroke, left and right pupil responses to light, and parenteral nutrition. The area under the receiver operator characteristic (ROC) curve of the prediction model constructed through these seven variables was 0.897 (95% CI: 0.876–0.919), and 0.896 (95% CI: 0.866–0.926) for the training and validation sets, respectively. Patients in the early tracheotomy group had a shorter length of hospital stay, IMV duration, and sedation duration compared to the late tracheotomy group (*p* < 0.05), but there was no statistically significant difference in survival outcomes between the two groups.

**Conclusion:**

The prediction model constructed and validated based on the MIMIC-IV database can accurately predict the outcome of tracheotomy in critically ill patients. Meanwhile, early tracheotomy in critically ill patients does not improve survival outcomes but has potential advantages in shortening the duration of hospitalization, IMV, and sedation.

**Supplementary Information:**

The online version contains supplementary material available at 10.1186/s12871-024-02558-x.

## Introduction

Invasive mechanical ventilation (IMV) is an important respiratory support technique. In the United States, 270–314 of every 100,000 people require IMV, and this number is increasing every year [1, 2]. In the intensive care unit (ICU), approximately 30–40% of patients require mechanical ventilation (IMV) [3]. Due to their condition, patients in the ICU use MV much more frequently and for a longer period than patients in the general ward. In patients requiring prolonged MV, further tracheotomy is often necessary [4]. Tracheotomy enhances patient oral care, decreases airway resistance, increases patient comfort, and decreases sedation medication use [5]. It has been suggested that early implementation of tracheotomy in mechanically ventilated patients can reduce the length of hospitalization, duration of invasive ventilation, and the incidence of ventilator-associated pneumonia [6–8]. Despite the many advantages of tracheotomy, its use as an invasive operation is generally limited to patients who are expected to require prolonged endotracheal intubation, given the risks and complications of the procedure. However, predicting the duration of invasive ventilation in clinical work is difficult. Moreover, for critically ill patients, prolonged endotracheal intubation not only aggravates airway injury but is more likely to induce lung infections and reduce the survival rate of patients [9].

Therefore, early identification of patients requiring tracheotomy in mechanically ventilated patients is of particular importance. This study retrospectively analyzed patients with successful extubation and tracheotomy in the Medical Information Marketplace in Intensive Care (MIMIC)-IV database to find risk factors for tracheotomy in critically ill patients. Constructing a prediction model to identify the high-risk group of tracheotomy at an early stage to guide clinical tracheotomy decision-making. Meanwhile, we analyze the prognostic differences of tracheotomy in different periods for tracheotomized patients and explore whether early tracheotomy is beneficial for critically ill patients.

## Materials and methods

### Research source

The data utilized in this research was sourced from the Medical Information Marketplace in Intensive Care IV database (MIMIC-IV, version 2.2) [10]. The MIMIC-IV database is made available largely through the work of researchers at the Massachusetts Institute of Technology Laboratory for Computational Physiology and collaborating research groups. The database contains information on more than 50,000 patients admitted to the emergency department or ICU within Beth Israel Deaconess Medical Center from 2008 to 2019. The database provides case information on patients throughout their hospitalization, including demographic data, vital signs, laboratory results, surgeries, medications, monitoring records, imaging reports, etc. All the patients in this database have been de-identified, so there are no ethical issues. Access to the database by researcher Xinghua Chen was granted following approval by the review boards of both the Massachusetts Institute of Technology and Beth Israel Deaconess Medical Center (Certificate No. 58951192).

### Subjects of the study

**Fig. 1:**
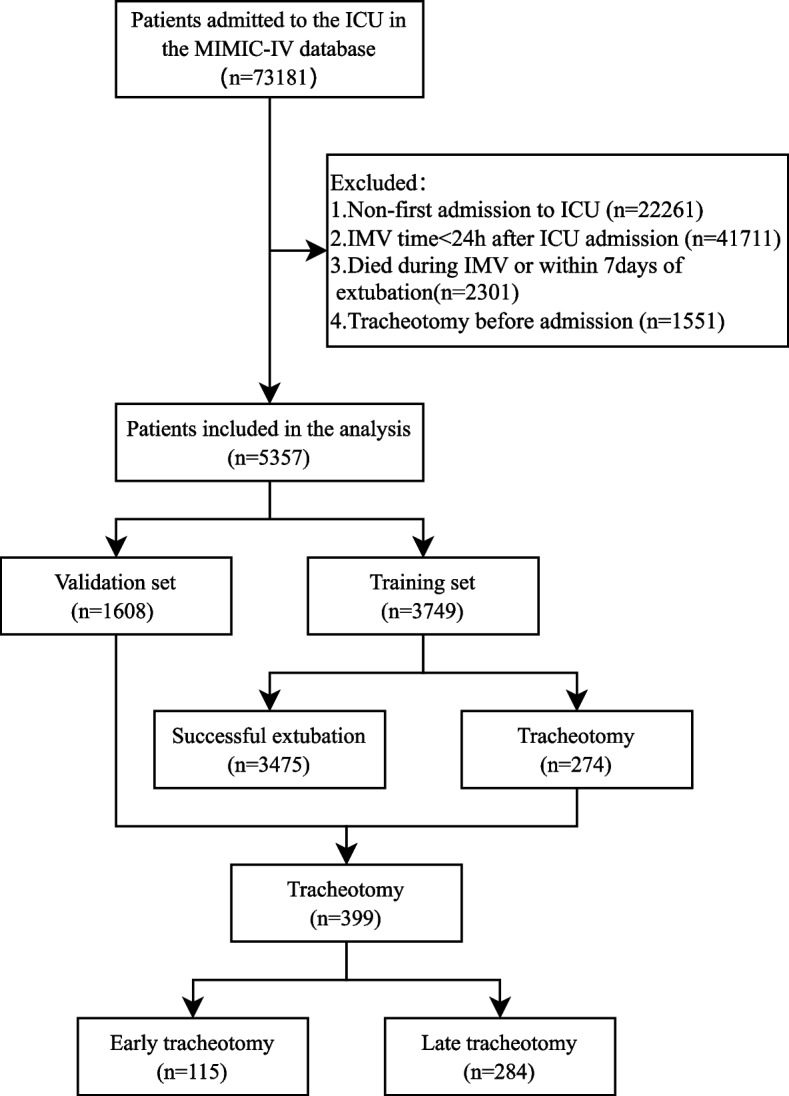
Flow chart

We collected clinical data from the MIMIC-IV database on patients who were admitted to the ICU for the first time and received more than 24 h of IMV after ICU admission. Patients were categorized into the successful extubation group and tracheotomy group according to whether they were subsequently successfully extubated or underwent tracheotomy. Successful extubation group was defined as extubation of the tracheal tube as planned and not reintubated during subsequent treatment, and survival within 7 days of extubation [11]. The tracheotomy group was defined as unsuccessful extubation of the tracheal tube with subsequent percutaneous tracheotomy or open tracheotomy. Exclusion of patients with tracheotomy before admission. Patients were randomized in a 7:3 ratio into training and validation sets. It is noted that only patients older than 18 years of age were included in the MIMIC-IV database. In addition, tracheotomized patients were categorized into an early tracheotomy group (< = 7 days) and a late tracheotomy group (> 7 days) based on the interval between the start of IMV and the implementation of tracheotomy. The flow chart of the study is shown in Fig. 1.

### Information collection

Retrospective collection of clinical data on critically ill patients. Including: (1) Demographic data: age, sex, body mass index (BMI); (2) Comorbidities: hypertension, diabetes, coronary artery disease (CAD), congestive heart failure (CHF), chronic obstructive pulmonary disease (COPD), traumatic intracerebral hemorrhage, hemorrhagic stroke, ischemic stroke, epilepsy, pneumonia, sepsis; (3) Various scores within 24 h of admission to the ICU: Acute Physiology Score-III (APS-III), Oxford Acute Severity of Illness Score (OASIS), Sequential Organ Failure Assessment (SOFA) score, Glasgow coma scale (GCS) score; (4) Laboratory tests within 24 h of ICU admission: red blood cell (RBC) count, white blood cell (WBC) count, platelet (PLT) count, hemoglobin (Hb), serum creatinine (SCr); (5) Vital signs within 24 h of ICU admission: temperature (T), heart rate (HR), systolic blood pressure (SBP), diastolic blood pressure (DBP), respiratory rate (RR), arterial oxygen saturation (SaO_2_), left pupil reaction to light, right pupil reaction to light; (6) Blood gas analysis within 24 h before extubation or tracheotomy: pH, lactic acids, partial pressure of oxygen (PO_2_), partial pressure of carbon dioxide (PCO_2_), oxygenation index (OI); (7) Treatment received during IMV: vasoactive drug therapy (VAT), continuous renal replacement therapy (CRRT); parenteral nutrition (PN).

In-hospital mortality, 90-day mortality, 1-year mortality, length of hospitalization, length of ICU stay, duration of IMV, and duration of sedative medication use were collected from tracheotomized patients. Patients ' comorbidity information was screened according to the international statistical classification of diseases and related health problems (ICD-9/10) in the patient's discharge diagnosis. All variables were missing within 20% of each other.

### Statistical analysis

Structured Query Language (SQL) and PostgreSQL tools were used to extract the medical records from the database, and STATA 15.1 and R 4.3.1 software were used to organize and analyze the data. Variables with less than 20% missing were filled by multiple interpolations using the MICE package of R software. Data were tested for normality using the Shapiro–Wilk test. Continuous variables following a normal distribution were presented as mean (standard deviation) and compared using the t-test. Non-normally distributed continuous variables were expressed as median (interquartile range) and compared using the Mann–Whitney U test. Categorical variables were presented as frequencies (proportions), with inter-group comparisons conducted using a chi-square test. Univariate logistic regression was used to find potential risk factors for tracheotomy in critically ill patients. Variables with *p*-values less than 0.2 in univariate logistic regression analyses were Lasso regression analyses and cross-validated to screen for variables with the most predictive value. Screened variables were included in a multifactorial logistic regression analysis to identify independent risk factors and construct predictive models. The predictive model was visualized by constructing a nomogram using R software, and dynamic web page nomograms were produced for clinical use. The Hosmer–Lemeshow test was used to evaluate the goodness of fit of the predictive model. The accuracy of the model in predicting tracheotomy outcomes was analyzed by plotting the receiver operator characteristic (ROC) curve and comparing the area under the ROC curve (AUC). Evaluating model accuracy by plotting calibration curves based on Bootstrap 1000 times self-service resampling. Decision curve analysis (DCA) was used to assess the clinical validity of the predictive model.

For patients with early and late tracheotomy, propensity score matching (PSM) was used to balance the difference between groups. A 1:1 nearest-neighbor matching technique was utilized to pair individuals with similar characteristics, with a caliper value of 0.1. The effectiveness of PSM was assessed by standardized mean difference (SMD), with SMD ≤  ± 0.1 indicating that the difference between the two groups was essentially equalized. We also performed a Kaplan–Meier analysis to assess 90-day and 1-year survival in patients with different timing of tracheotomy and assessed differences between groups by log-rank test. All tests were two-sided, and *p* < 0.05 was considered a statistically significant difference.

## Result

### Comparison of baseline information for the training set

A total of 5357 patients were enrolled in the study and randomized in a 7:3 ratio into a training set (*n* = 3749) and a validation set (*n* = 1608). A total of 3749 patients were enrolled in the training set, of whom 3475 were successfully extubated and 274 were tracheotomized. The validation set had a total of 1608 patients enrolled in the study, of which 1483 were successfully extubated and 125 were tracheotomized. Baseline characteristics of patients in the training set are shown in Table 1. The results showed that there were statistically significant differences between the successful extubation group and the tracheotomy group in terms of BMI, CAD, CHF, COPD, traumatic intracerebral hemorrhage, hemorrhagic stroke, pneumonia, APS-III, OASIS, GCS score, WBC count, PLT count, Hb, SCr, SBP, RR, left and right pupil responses to light, pH, PCO_2_, VAT and PN (all *p* < 0.05). The baseline characteristics of the total population, training set, and validation set of patients are shown in Supplementary Table 1. The results showed that the differences in most variables were not statistically significant (*p* > 0.05), except for differences between the training and validation sets on RBC count and WBC count.

**Table 1:** Baseline characteristics of patients in the training set

Variables	Successful extubation group (*n* = 3475)	Tracheotomy group (*n* = 274)	*p-*value
Demographic, median (IQR)			
Age, years	64.5 (52.4 to 75.6)	62.7 (52.0 to 74.2)	.254
Sex, male, n(%)	1989 (57.2%)	174 (63.5%)	.050
BMI, kg/m^2^	27.7 (23.9 to 32.7)	26.6 (23.5 to 31.6)	.019
Comorbidity, n (%)			
Hypertension	979 (28.2%)	92 (33.6%)	.066
Diabetes	998 (28.7%)	73 (26.6%)	.507
CAD	888 (25.6%)	49 (17.9%)	.006
CHF	552 (15.9%)	28 (10.2%)	.016
COPD	212 (6.1%)	10 (3.6%)	.128
Traumatic intracerebral hemorrhage	140 (4%)	28 (10.2%)	< .001
Hemorrhagic stroke	305 (8.8%)	55 (20.1%)	< .001
Ischemic stroke	196 (5.6%)	23 (8.4%)	.082
Epilepsy	178 (5.1%)	21 (7.7%)	.096
Pneumonia	1658 (47.7%)	221 (80.7%)	< .001
Sepsis	838 (24.1%)	67 (24.5%)	.958
Various scores, median (IQR)			
APSIII	56.0 (41.0 to 75.0)	69.0 (52.0 to 91.0)	< .001
OASIS	39.0 (34.0 to 44.0)	40.0 (35.0 to 47.0)	< .001
SOFA score	7.0 (5.0 to 10.0)	7.0 (5.0 to 11.0)	.999
GCS score	11.0 (8.0 to 14.0)	7.0 (3.0 to 10.0)	< .001
Laboratory tests, median (IQR)			
RBC count, × 10^12^/L	3.9 (3.3 to 4.4)	4.0 (3.4 to 4.4)	.082
WBC count, × 10^9^/L	11.2 (7.9 to 15.6)	12.0 (9.1 to 16.4)	.021
PLT count, × 10^9^/L	213.0 (159.5 to 281.0)	234.0 (182.0 to 278.0)	.011
Hb, g/dl	11.7 (9.8 to 13.4)	12.1 (10.4 to 13.4)	.059
Scr, mg/dl	1.0 (0.8 to 1.5)	0.9 (0.7 to 1.3)	.001
Vital signs, median (IQR)			
T, ℃	37.1 (36.7 to 37.4)	37.1 (36.7 to 37.5)	.235
HR, n/min	85.2 (75.4 to 98.1)	83.4 (75.0 to 96.7)	.498
SBP, mmHg	112.4 (103.2 to 124.9)	117.0 (106.0 to 131.7)	< .001
DBP, mmHg	62.9 (55.6 to 70.7)	64.0 (56.2 to 72.3)	.162
RR, n/min	18.6 (16.4 to 21.5)	19.3 (17.2 to 22.1)	.009
SaO_2_, %	98.0 (96.5 to 99.2)	98.2 (96.6 to 99.4)	.092
Left pupil reaction to light, n (%)			< .001
Brisk	3308 (95.2%)	96 (35%)	
Sluggish	137 (3.9%)	122 (44.5%)	
Non-reactive	30 (0.9%)	56 (20.4%)	
Right pupil reaction to light, n (%)			< .001
Brisk	3308 (95.2%)	107 (39.1%)	
Sluggish	137 (3.9%)	114 (41.6%)	
Non-reactive	30 (0.9%)	53 (19.3%)	
Blood gas analysis, median (IQR)			
Ph	7.4 (7.4 to 7.5)	7.4 (7.4 to 7.5)	< .001
Lactic acids, mmol/L	1.6 (1.1 to 2.5)	1.6 (1.1 to 2.3)	.451
PO_2,_ mmHg	107.0 (81.0 to 134.0)	113.0 (91.0 to 147.0)	.001
PCO_2,_ mmHg	41.0 (36.0 to 46.0)	40.0 (35.0 to 45.0)	.165
IO, mmHg	260.0 (187.5 to 336.0)	262.2 (196.0 to 343.3)	.253
Treatment received, n (%)			
VAT	2140 (61.6%)	191 (69.7%)	.009
CRRT	228 (6.6%)	24 (8.8%)	.203
PN	160 (4.6%)	34 (12.4%)	< .001

*Abbreviations*: *IQR* interquartile range, *BMI* body mass index, *CAD* coronary artery disease, *CHF* congestive heart failure, *COPD* chronic obstructive pulmonary disease, *APSIII* acute physiology scoreIII, *OASIS* Oxford Acute Severity of Illness Score, *SOFA* sequential organ failure assessment, *GCS* glasgow coma scale, *RBC* red blood cell, *WBC* white blood cell, *PLT* platelet, *Hb* hemoglobin, *SCr* serum creatinine, *T* temperature, *HR* heart rate, *SBP* systolic blood pressure, *DBP* diastolic blood pressure, *RR* respiratory rate, *SaO*_*2*_ arterial oxygen saturation, *PO*_*2*_ partial pressure of oxygen, *PCO*_*2*_ partial pressure of carbon dioxide, *OI* oxygenation index, *VAT* vasoactive drug therapy, *CRRT* continuous renal replacement therapy, *PN* parenteral nutrition

### Screening of predictors

**Fig. 2:**
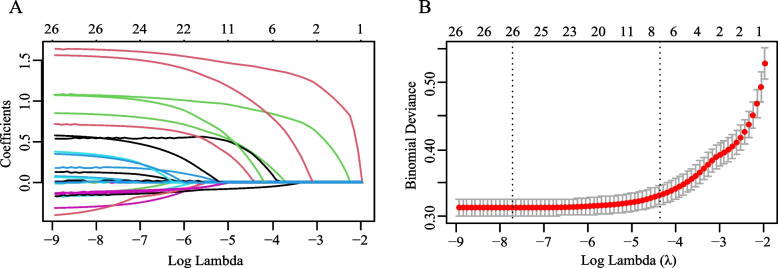
Lasso regression screening variables: Figure **A** shows the path of the Lasso coefficients for the 26 risk factors; Figure **B** shows the cross-validation curves of the Lasso regression showing the course of the optimal penalty term (λ). The dashed line on the left shows λ (λmin) at the smallest deviation, and the dashed line on the right shows λmin + 1 standard error

Univariate logistic regression analysis was performed for the training group (Supplementary Table 2). To miss as few valuable variables as possible, we included variables with *p* less than 0.2 in the univariate logistic regression analysis in the Lasso regression analyses and cross-validated them (Fig. 2). To prevent overfitting of the model and to make the predictive model as concise as possible, we select the final candidate variables with a penalty term (λ) = λmin + 1 standard errors. Seven variables predicting tracheotomy in critically ill patients were finally screened: GCS score, pneumonia, traumatic intracerebral hemorrhage, hemorrhagic stroke, left and right pupil responses to light, and PN. These seven variables were included in a multifactorial logistic regression model, and the results showed that they were all independent risk factors for tracheotomy in critically ill patients(all *p* < 0.05, Table 2).

**Table 2:** Multifactor logistic regression analysis of variables screened in the training set

Variables	Coefficients	OR	95%CI	*p-*value
GCS score	-0.126	0.882	0.846–0.919	< 0.001
Pneumonia	0.952	2.591	1.851–3.750	0.001
Traumatic intracerebral hemorrhage	1.036	2.818	1.599–4.818	0.001
Hemorrhagic stroke	0.956	2.600	1.694–3.947	< 0.001
Left pupil reaction to light				
Brisk		Ref	Ref	
Sluggish	2.498	12.164	6.499–23.781	< 0.001
Non-reactive	2.705	14.960	6.462–34.560	< 0.001
Right pupil reaction to light				
Brisk		Ref	Ref	
Sluggish	0.791	2.206	1.123–4.344	0.065
Non-reactive	1.487	4.425	1.845–10.541	< 0.001
PN	1.199	3.318	1.904–5.623	< 0.001

*Abbreviations*: *GCS* glasgow coma scale, *PN* parenteral nutrition

### Construction of nomogram prediction model

**Fig. 3:**
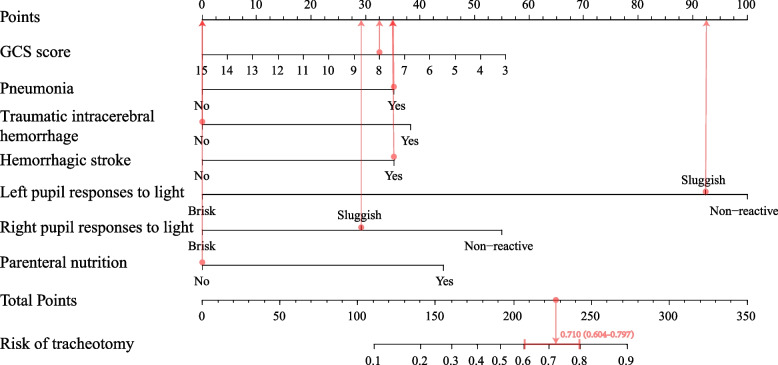
Nomogram: Each variable is scored from 0 to 100. A vertical line is drawn on the axis as a score according to the particular state the variable is in. The scores for each variable are summed to give a total score, based on which to assess the patient's risk of tracheotomy

Based on multifactorial logistic regression analysis, GCS score, pneumonia, traumatic intracerebral hemorrhage, hemorrhagic stroke, and left and right pupil responses to light and PN, were selected to establish a prediction model for tracheotomy in critically ill patients in the present study and plotted nomogram (Fig. 3). Each important variable in the graph is assigned a weighted score from 0 to 100, and a total score is calculated by summing the scores for each risk factor in the nomogram to accurately predict the risk of tracheotomy in critically ill patients. The higher the total score, the higher the risk of tracheotomy. To facilitate clinical applications, the DynNom package of R software was used to create online dynamic nomograms (https://chentracheotomy.shinyapps.io/dynnomapp/).

### Evaluation and validation of predictive models

**Fig. 4:**
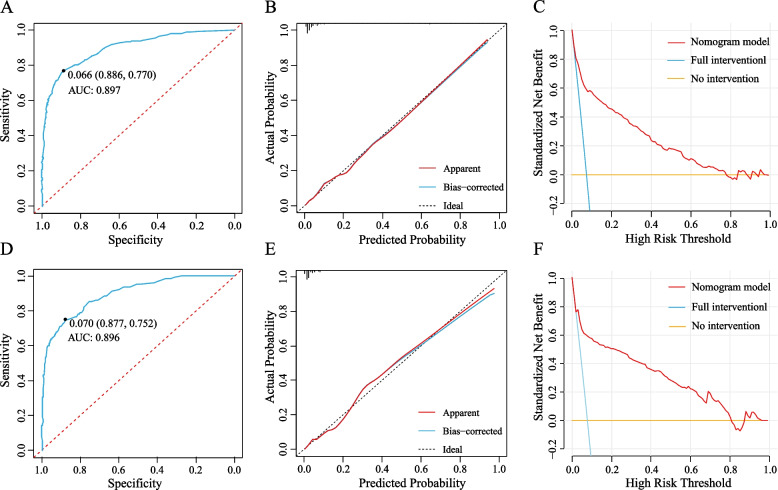
Evaluation and validation of predictive models: Figures **A** and **D** show the ROC curves for the training and validation sets, Figures **B** and **D** show the calibration curves for the training and validation sets, and Figures **C** and F show the DCA curves

The Hosmer–Lemeshow test results for the prediction model in the training and validation sets are χ^2^ = 11.134 (*p* = 0.194 > 0.05) and χ^2^ = 4.293 (*p* = 0.830 > 0.05), which demonstrate that the model has good goodness-of-fit in both datasets. The evaluation and validation results of the prediction model are shown in Fig. 4. The AUC of the training set was 0.897 (95% CI: 0.876–0.919), and the sensitivity and specificity of the prediction model were 88.6% and 77.0%, respectively, when the critical value was the maximum value of the Youden index. The AUC of the validation set was 0.896 (95% CI: 0.866–0.926), and the sensitivity and specificity of the prediction model were 87.7% and 75.2%, respectively, when the critical value was the maximum value of the Youden index. It indicates that the prediction model showed excellent prediction ability in both groups of patients. The calibration curves plotted are all close to the reference line, indicating good agreement between predicted and observed outcomes. The results of the DCA showed that in the training and validation sets when the threshold probability of tracheotomy in critically ill patients was in the range of 0.02 to 0.78 and 0.03 to 0.81, the level of net benefit of applying the prediction model was significantly higher than that of the "no-intervention" and "full intervention" program. This suggests that the prediction model has good clinical applicability.

### Comparison of baseline data for early and late tracheotomy before and after PSM

Before PSM there were statistically significant differences in sex, CAD, pneumonia, sepsis, APS-III, OASIS, SOFA score, GCS score, SaO_2_, PO_2_, OI, VAT, CRRT, and PN between the two groups of patients with tracheotomy (all* p* < 0.05, Table 3). After PSM there were no statistically significant differences in baseline characteristics between the two groups of patients (all *p* > 0.05, Supplementary Table 3). The difference in SMD between the two groups for most of the variables after PSM was less than ± 0.1, indicating that PSM had effectively reduced the differences between groups (Supplementary Fig. 1).

**Table 3:** Baseline characteristics of patients in the early and late tracheotomy groups before PSM

Variables	Total (*n* = 399)	Early tracheotomy (*n* = 115)	Late tracheotomy (*n* = 284)	*p-*value
Demographic, median (IQR)				
Age, years	65.8 (53.9 to 75.6)	62.7 (44.7 to 74.6)	66.7 (56.2 to 75.7)	.065
Sex, male, n(%)	246 (61.7%)	81 (70.4%)	165 (58.1%)	.029
BMI, kg/m^2^,	26.8 (23.9 to 31.8)	27.0 (23.9 to 31.2)	26.8 (23.9 to 32.4)	.833
Comorbidity, n (%)				
Hypertension	145 (36.3%)	43 (37.4%)	102 (35.9%)	.871
Diabetes	111 (27.8%)	34 (29.6%)	77 (27.1%)	.710
CAD	83 (20.8%)	15 (13%)	68 (23.9%)	.022
CHF	52 (13%)	12 (10.4%)	40 (14.1%)	.414
COPD	14 (3.5%)	2 (1.7%)	12 (4.2%)	.356
Traumatic intracerebral hemorrhage	40 (10%)	17 (14.8%)	23 (8.1%)	.067
Hemorrhagic stroke	91 (22.8%)	22 (19.1%)	69 (24.3%)	.326
Ischemic stroke	31 (7.8%)	6 (5.2%)	25 (8.8%)	.315
Epilepsy	28 (7%)	4 (3.5%)	24 (8.5%)	.122
Pneumonia	323 (81%)	84 (73%)	239 (84.2%)	016
Sepsis	102 (25.6%)	15 (13%)	87 (30.6%)	< .001
Various scores, median (IQR)				
APSIII	69.0 (53.0 to 90.0)	60.0 (43.0 to 77.0)	71.0 (58.0 to 93.0)	< .001
OASIS	41.6 ± 8.0	39.1 ± 7.7	42.6 ± 7.9	< .001
SOFA score	7.0 (5.0 to 10.0)	6.0 (5.0 to 9.0)	7.0 (5.0 to 11.0)	< .001
GCS score	7.0 (3.0 to 10.0)	8.0 (6.0 to 11.0)	6.5 (3.0 to 9.0)	< .001
Laboratory tests, median (IQR)				
RBC count, × 10^12^/L	3.9 ± 0.8	3.9 ± 0.7	3.9 ± 0.8	.982
WBC count, × 10^9^/L	12.0 (8.9 to 16.8)	12.5 (9.3 to 16.8)	11.6 (8.4 to 16.6)	.416
PLT count, × 10^9^/L	226.0 (176.0 to 281.5)	227.0 (183.5 to 283.5)	226.0 (173.0 to 279.5)	.576
Hb, g/dl	12.0 (10.3 to 13.5)	12.1 (10.2 to 13.4)	11.9 (10.3 to 13.6)	.716
Scr, mg/dl	1.0 (0.7 to 1.3)	0.9 (0.7 to 1.2)	1.0 (0.7 to 1.4)	.524
Vital signs, median (IQR)				
T, ℃	37.1 (36.8 to 37.5)	37.1 (36.7 to 37.6)	37.2 (36.8 to 37.5)	.951
HR, n/min	83.5 (74.5 to 96.4)	80.9 (72.9 to 94.8)	84.6 (75.2 to 97.1)	.124
SBP, mmHg	117.1 (106.1 to 130.3)	118.6 (106.5 to 130.8)	116.5 (105.6 to 130.1)	.753
DBP, mmHg	63.1 (55.5 to 72.4)	62.1 (54.8 to 69.2)	64.0 (56.0 to 73.0)	.197
RR, n/min	19.3 (17.1 to 22.2)	18.8 (16.7 to 21.3)	19.5 (17.4 to 22.7)	.057
SaO_2_, %	98.2 (96.4 to 99.4)	98.9 (97.0 to 99.6)	97.9 (96.2 to 99.1)	.002
Left pupil reaction to light, n (%)				.188
Brisk	143 (35.8%)	42 (36.5%)	101 (35.6%)	
Sluggish	179 (44.9%)	45 (39.1%)	134 (47.2%)	
Non-reactive	77 (19.3%)	28 (24.3%)	49 (17.3%)	
Right pupil reaction to light, n (%)				.453
Brisk	153 (38.3%)	45 (39.1%)	108 (38%)	
Sluggish	169 (42.4%)	44 (38.3%)	125 (44%)	
Non-reactive	77 (19.3%)	26 (22.6%)	51 (18%)	
Blood gas analysis, median (IQR)				
Ph	7.4 (7.4 to 7.5)	7.4 (7.4 to 7.5)	7.4 (7.4 to 7.5)	.288
Lactic acids, mmol/L	1.6 (1.1 to 2.4)	1.5 (1.0 to 2.5)	1.6 (1.1 to 2.3)	.408
PO_2,_ mmHg	112.0 (87.0 to 139.5)	121.0 (97.0 to 161.0)	108.0 (82.5 to 131.0)	< .001
PCO_2,_ mmHg	41.0 (35.0 to 46.0)	40.0 (35.0 to 46.0)	41.0 (35.0 to 46.0)	.937
IO, mmHg	262.0 (190.0 to 340.0)	288.3 (215.0 to 384.2)	252.9 (185.0 to 324.2)	.003
Treatment received, n (%)				
VAT	284 (71.2%)	71 (61.7%)	213 (75%)	.012
CRRT	41 (10.3%)	1 (0.9%)	40 (14.1%)	< .001
PN	47 (11.8%)	2 (1.7%)	45 (15.8%)	< .001

*Abbreviations*: *IQR* interquartile range, *BMI* body mass index, *CAD* coronary artery disease, *CHF* congestive heart failure, *COPD* chronic obstructive pulmonary disease, *APSIII* acute physiology scoreIII, *OASIS* Oxford Acute Severity of Illness Score, *SOFA* sequential organ failure assessment, *GCS* glasgow coma scale, *RBC* red blood cell, *WBC* white blood cell, *PLT* platelet, *Hb* hemoglobin, *SCr* serum creatinine, *T* temperature, *HR* heart rate, *SBP* systolic blood pressure, *DBP* diastolic blood pressure, *RR* respiratory rate, *SaO*_*2*_ arterial oxygen saturation, *PO*_*2*_ partial pressure of oxygen, *PCO*_*2*_ partial pressure of carbon dioxide, *OI* oxygenation index, *VAT* vasoactive drug therapy, *CRRT* continuous renal replacement therapy, *PN* parenteral nutrition therapy

### Comparison of outcomes in patients with early and late tracheotomy

**Fig. 5:**
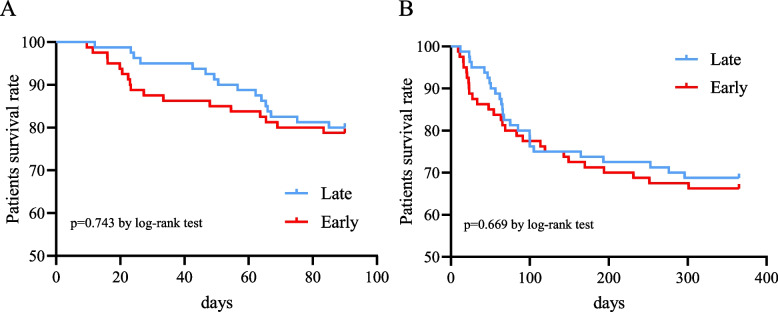
Kaplan–Meier’s survival analysis of the 90-day (**A**) and 1-year (**B**)

In the post-PSM cohort, the differences in in-hospital mortality (10% vs 8.8%), 90-day mortality (21.2% vs 20%), and 1-year mortality (33.8% vs 31.2%) between the early and late tracheotomy groups were not statistically significant (all *p* > 0.05, Table 4). The length of hospitalization (18.2 [14.1, 26.6] vs 24.6 [18.1, 33.6] days), ICU stay (12.3 [8.9, 16.1] vs 19.1 [13.4, 24.5] days), IMV (10 [6.7, 14.2] vs 16.6 [12.4, 21.9] days), and sedation (8.0 [6.0, 11.5] vs 14.0 [11.0, 18.0] days) in the early tracheotomy group were shorter than those in the late tracheotomy group, and the difference was statistically significant (all *p* < 0.05). However, the difference in IMV duration after tracheotomy between the two groups was not statistically significant (*p* > 0.05). The Kaplan–Meier curves for the two groups of patients after PSM are shown in Fig. 5. There were no statistically significant differences between groups in 90-day survival and 1-year survival rates after the log-rank test between the two groups of patients (both* p* > 0.05).

**Table 4:** Outcomes of early and late tracheotomy in the cohort after PSM

Outcomes	Total (*n* = 152)	Early tracheotomy (*n* = 76)	Late tracheotomy (*n* = 76)	*p*- value
In-hospital mortality, n (%)	15 (9.4%)	8 (10%)	7 (8.8%)	1.000
90-day mortality, n (%)	33 (20.6%)	17 (21.2%)	16 (20%)	1.000
1-year mortality, n (%)	52 (32.5%)	27 (33.8%)	25 (31.2%)	.866
Hospitalization time, days, median (IQR)	21.6 (16.0 to 30.7)	18.2 (14.1 to 26.6)	24.6 (18.1 to 33.9)	< .001
Length of ICU stay, days, median (IQR)	15.0 (11.1 to 20.9)	12.3 (8.9 to 16.1)	19.1 (13.4 to 24.5)	< .001
Duration of IMV, days, median (IQR)	13.2 (9.4 to 18.3)	10.0 (6.7 to 14.2)	16.6 (12.4 to 21.9)	< .001
Duration of IMV after tracheotomy, days, median (IQR)	4.1 (2.0 to 8.1)	5.1 (2.0 to 9.2)	3.5 (2.0 to 7.3)	.269
Duration of sedation, days, median (IQR)	11.0 (8.0 to 16.0)	8.0 (6.0 to 11.0)	14.0 (11.0 to 18.0)	< .001

*Abbreviations*: *IQR* interquartile range, *ICU* intensive care unit, *IMV* Invasive mechanical ventilation

## Discussion

In this study, using a large sample of critically ill patients from the MIMIC-IV database, we successfully developed a prediction model with seven simple indicators: GCS score, pneumonia, traumatic intracerebral hemorrhage, hemorrhagic stroke, left and right pupil responses to light and PN. The predictive model showed good predictive efficacy and clinical validity in both the training and validation sets, which is a significant guide for clinicians in making tracheotomy decisions.

We found that patients with pneumonia were more likely to develop tracheotomy. A large retrospective cohort study showed that pneumonia was the most important cause of prolonged mechanical ventilation in critically ill patients [12]. Pneumonia, in addition to direct damage to the lung parenchyma caused by pathogens, also causes inflammation and edema of the lung tissue, affecting alveolar gas exchange and leading to respiratory failure [13, 14]. The study by Peñuelas et al. also found that patients with pneumonia were more likely to present with extubation failure and prolonged mechanical ventilation [15]. Similarly, the creation of an artificial airway removes the protective effect of the epiglottis and airway mucosa, making it easier for external or oral bacteria to enter the lower airways and exacerbate lung infections. Many studies have found that the longer the duration of mechanical ventilation, the higher the risk of ventilator-associated pneumonia [16–18].

We also found that patients with neurologic compromise were at higher risk of tracheotomy. This is mainly due to the fact that damage to the central nervous system can affect the regulation of the respiratory center, including respiratory rate and depth, airway tone, as well as normal neural reflexes, including the cough reflex, swallowing reflex, apnea, and respiratory facilitation [19]. Meanwhile, severe neurologic impairment is often accompanied by feeding disorders, so we found that patients who received PN were more likely to have a subsequent tracheotomy. In ICU patients, the long-term inability to feed themselves orally is more likely to lead to malnutrition [20]. Chronic malnutrition reduces respiratory muscle mass and compromises respiratory effectiveness [21, 22]. A study by Chuang CY et al. also noted that underweight patients were at a higher risk of extubation failure compared to normal-weight and overweight patients [23]. In addition, the adverse effects of neurologic impairment in critically ill patients are not limited to prolonged ventilation. A prospective cohort study of acute stroke patients in a neurointensive care unit found that higher National Institutes of Health Stroke Scale (NIHSS) scores on admission were associated with a worse prognosis for patients at 90 and 180 days [24]. Perhaps our predictive model has more promising applications in neurointensive care units.

In addition, our team compared the prognosis of critically ill patients who underwent tracheotomy at different times. After utilizing propensity score matching to reduce the effect of confounding factors, we found that early and late tracheotomy groups did not show statistically significant differences in in-hospital mortality, 90-day mortality, and 1-year mortality. There was a statistically significant shortening of the early tracheotomy group for the late tracheotomy group in terms of length of hospitalization, ICU stay, IMV time, and sedation time. Many previous studies have produced similar results [6, 25, 26]. This is probably related to the fact that tracheotomy increases patient comfort and reduces the need for sedation [5]. Shorter sedation times allow patients to regain voluntary respiration, swallowing, and communication functions more quickly and help patients discontinue invasive ventilation. It also allows for a higher level of consciousness so that patients can get out of bed early, which helps them recover early. In addition, sustained sedation has been associated with many side effects, including hypotension, respiratory depression, and impaired cognition [27, 28]. Prolonged mechanical ventilation and sedation are associated with an increased incidence of ICU-acquired weakness, which is more likely to be avoided by early tracheotomy [29].

There are some limitations to this study: first, we were unable to include some potentially valuable variables, such as data on respiratory mechanics and ventilator parameter settings, due to the high level of missingness of some variables; and second, in the case of patients with pneumonia, we were unable to determine whether it was community-acquired or hospital-acquired pneumonia. Finally, this study is a retrospective study and the conclusions drawn need to be tested and refined in prospective cohort studies.

## Conclusions

Based on a large intensive care database, MIMIC-IV, we developed and validated a prediction model for tracheotomy in critically ill patients. Based on this prediction model, the risk of performing tracheotomy in critically ill patients can be effectively predicted at an early stage and guided to make tracheotomy decisions. However, for critically ill patients, although early tracheotomy has many advantages, such as shorter hospitalization time, invasive ventilation time, and sedation time, it does not show advantages in reducing patient mortality.

### Supplementary Information

Supplementary Material 1.

## Data Availability

The datasets generated and analyzed during this study can be accessed from the MIMIC-IV database at https://mimic.physionet.org/iv/, or obtained from the corresponding author upon reasonable request.

## Abbreviations

ICU: Intensive care unit
IMV: Invasive mechanical ventilation
MV: Mechanical ventilation
MIMIC-IV: Medical information mart for intensive care IV
BMI: Body mass index
CAD: Coronary artery disease
CHF: Congestive heart failure
COPD: Chronic obstructive pulmonary disease
APS-III: Acute physiology score-III
OASIS: Oxford acute severity of illness score
SOFA: Sequential organ failure assessment
GCS: Glasgow coma scale
SBP: Systolic blood pressure
DBP: Diastolic blood pressure
OI: Oxygenation index
VAT: Vasoactive drug therapy
CRRT: Continuous renal replacement therapy
PN: Parenteral nutrition
ICD: The International Statistical Classification of Diseases and Related Health Problems
SQL: Structured query language
ROC: Receiver operator characteristic
AUC: The area under the ROC curve
DCA: Decision curve analysis
PSM: Propensity score matching
SMD: Standardized mean difference
