# Supplementary material for: Establishment and validation of a predictive model for tracheotomy in critically ill patients and analysis of the impact of different tracheotomy timing on patient prognosis
Source: BMC Anesthesiol. 2024 May 17;24:175. doi: 10.1186/s12871-024-02558-x (PMC11100231; doi:10.1186/s12871-024-02558-x)
Supplement: Supplementary file 1 — Supplementary Material 1. [file 12871_2024_2558_MOESM1_ESM.docx]

**Supplementary file**

**Supplementary Table 1.** Baseline characteristics of patients in the total population, training set, and validation set.

| Variables | Total  (n=5357) | | Training set  (n=3749) | Validation set  (n=1608) | *p-*value |
| --- | --- | --- | --- | --- | --- |
| Successful extubation, n (%) | 4958 (92.6%) | | 3475 (92.7%) | 1483 (92.2%) | .591 |
| Tracheotomy, n (%) | 399 (7.4%) | | 274 (7.3%) | 125 (7.8%) |  |
| Demographic, median (IQR) | |  |  |  |  |
| Age, years | 64.5 (52.6 to 75.7) | | 64.4 (52.4 to 75.5) | 65.0 (52.8 to 76.3) | .418 |
| Sex, male, n(%) | 3094 (57.8%) | | 2163 (57.7%) | 931 (57.9%) | .914 |
| BMI, kg/m^2^, | 27.7 (23.8 to 32.7) | | 27.6 (23.8 to 32.6) | 28.0 (23.8 to 33.0) | .370 |
| Comorbidity, n (%) | |  |  |  |  |
| Hypertension | 1525 (28.5%) | | 1071 (28.6%) | 454 (28.2%) | .830 |
| Diabetes | 1522 (28.4%) | | 1071 (28.6%) | 451 (28%) | .723 |
| CAD | 1356 (25.3%) | | 937 (25%) | 419 (26.1%) | .432 |
| CHF | 840 (15.7%) | | 580 (15.5%) | 260 (16.2%) | .546 |
| COPD | 325 (6.1%) | | 222 (5.9%) | 103 (6.4%) | .537 |
| Traumatic intracerebral hemorrhage | 241 (4.5%) | | 168 (4.5%) | 73 (4.5%) | .982 |
| Hemorrhagic stroke | 535 (10%) | | 360 (9.6%) | 175 (10.9%) | .167 |
| Ischemic stroke | 331 (6.2%) | | 219 (5.8%) | 112 (7%) | .133 |
| Epilepsy | 298 (5.6%) | | 199 (5.3%) | 99 (6.2%) | .239 |
| Pneumonia | 2664 (49.7%) | | 1879 (50.1%) | 785 (48.8%) | .399 |
| Sepsis | 1299 (24.2%) | | 905 (24.1%) | 394 (24.5%) | .803 |
| Various scores, median (IQR) | |  |  |  |  |
| APSⅢ | 57.0 (41.0 to 76.0) | | 57.0 (42.0 to 76.0) | 56.0 (41.0 to 75.0) | .410 |
| OASIS | 39.0 (34.0 to 44.0) | | 39.0 (34.0 to 44.0) | 39.0 (34.0 to 45.0) | .822 |
| SOFA score | 7.0 (5.0 to 10.0) | | 7.0 (5.0 to 10.0) | 7.0 (5.0 to 10.0) | .527 |
| GCS score | 11.0 (7.0 to 14.0) | | 11.0 (7.0 to 14.0) | 11.0 (7.0 to 14.0) | .435 |
| Laboratory tests, median (IQR) | |  |  |  |  |
| RBC count, ×10^12^/L | 3.9 (3.3 to 4.5) | | 3.9 (3.3 to 4.4) | 4.0 (3.4 to 4.5) | .029 |
| WBC count, ×10^9^/L | 11.4 (8.1 to 15.8) | | 11.3 (8.0 to 15.6) | 11.6 (8.3 to 16.1) | .031 |
| PLT count, ×10^9^/L | 215.0 (161.0 to 281.0) | | 214.0 (161.0 to 281.0) | 217.0 (161.0 to 280.5) | .696 |
| Hb, g/dl | 11.7 (9.9 to 13.4) | | 11.7 (9.8 to 13.4) | 11.8 (10.0 to 13.5) | .066 |
| Scr, mg/dl | 1.0 (0.8 to 1.5) | | 1.0 (0.8 to 1.5) | 1.0 (0.8 to 1.5) | .927 |
| Vital signs, median (IQR) | |  |  |  |  |
| T, ℃ | 37.1 (36.7 to 37.4) | | 37.1 (36.7 to 37.4) | 37.1 (36.7 to 37.4) | .671 |
| HR, n/min | 85.1 (75.1 to 98.0) | | 85.1 (75.2 to 98.0) | 85.1 (74.5 to 97.6) | .695 |
| SBP, mmHg | 112.5 (103.2 to 125.3) | | 112.7 (103.4 to 125.3) | 112.1 (102.9 to 125.2) | .434 |
| DBP, mmHg | 62.9 (55.7 to 70.8) | | 63.0 (55.6 to 70.8) | 62.9 (55.7 to 70.8) | .902 |
| RR, n/min | 18.7 (16.5 to 21.6) | | 18.7 (16.4 to 21.5) | 18.8 (16.6 to 21.7) | .198 |
| SaO_2_, % | 98.0 (96.5 to 99.2) | | 98.0 (96.5 to 99.2) | 98.1 (96.3 to 99.2) | .584 |
| Left pupil reaction to light, n (%) | | | | | .322 |
| Brisk | 4854 (90.6%) | | 3404 (90.8%) | 1450 (90.2%) |  |
| Sluggish | 386 (7.2%) | | 259 (6.9%) | 127 (7.9%) |  |
| Non-reactive | 117 (2.2%) | | 86 (2.3%) | 31 (1.9%) |  |
| Right pupil reaction to light, n (%) | | | | | .356 |
| Brisk | 4860 (90.7%) | | 3415 (91.1%) | 1445 (89.9%) |  |
| Sluggish | 375 (7%) | | 251 (6.7%) | 124 (7.7%) |  |
| Non-reactive | 122 (2.3%) | | 83 (2.2%) | 39 (2.4%) |  |
| Blood gas analysis, median (IQR) | |  |  |  |  |
| Ph | 7.4 (7.4 to 7.5) | | 7.4 (7.4 to 7.5) | 7.4 (7.4 to 7.5) | .894 |
| Lactic acids, mmol/L | 1.6 (1.1 to 2.5) | | 1.6 (1.1 to 2.5) | 1.7 (1.2 to 2.6) | .232 |
| PO_2,_ mmHg | 106.0 (81.0 to 134.0) | | 107.0 (82.0 to 135.0) | 105.0 (81.0 to 132.0) | .088 |
| PCO_2,_ mmHg | 41.0 (36.0 to 46.0) | | 41.0 (36.0 to 46.0) | 41.0 (36.0 to 46.0) | .791 |
| IO, mmHg | 260.0 (188.0 to 333.3) | | 262.0 (188.0 to 336.7) | 255.0 (187.5 to 330.0) | .182 |
| Treatment received, n (%) | |  |  |  |  |
| VAT | 3366 (62.8%) | | 2331 (62.2%) | 1035 (64.4%) | .137 |
| CRRT | 375 (7%) | | 252 (6.7%) | 123 (7.6%) | .246 |
| PN | 280 (5.2%) | | 194 (5.2%) | 86 (5.3%) | .846 |

Abbreviations: IQR, interquartile range; BMI, body mass index; CAD, coronary artery disease; CHF, congestive heart failure; COPD, chronic obstructive pulmonary disease; APSⅢ, acute physiology scoreⅢ; OASIS, Oxford Acute Severity of Illness Score; SOFA, sequential organ failure assessment; GCS, glasgow coma scale; RBC, red blood cell; WBC, white blood cell; PLT, platelet; Hb, hemoglobin; SCr, serum creatinine; T, temperature; HR, heart rate; SBP, systolic blood pressure; DBP, diastolic blood pressure; RR, respiratory rate; SaO_2_, arterial oxygen saturation; PO_2_, partial pressure of oxygen; PCO_2_, partial pressure of carbon dioxide; OI, oxygenation index; VAT, vasoactive drug therapy; CRRT, continuous renal replacement therapy; PN, parenteral nutrition.

**Supplementary Table 2.** Univariate logistic regression analysis for each variable in the training set.

| Variables | OR | | 95%CI | *p-*value |
| --- | --- | --- | --- | --- |
| Demographic | |  |  |  |
| Age | .995 | | 0.988-1.003 | .222 |
| Sex | 1.3 | | 1.007-1.678 | .044 |
| BMI | .979 | | 0.953-0.987 | .001 |
| Comorbidity | |  |  |  |
| Hypertension | 1.289 | | 0.992-1.674 | .057 |
| Diabetes | .901 | | 0.683-1.19 | .464 |
| CAD | .634 | | 0.462-0.872 | .005 |
| CHF | .603 | | 0.403-0.9 | .013 |
| COPD | .583 | | 0.305-1.113 | .102 |
| Traumatic intracerebral hemorrhage | 2.711 | | 1.771-4.151 | <0.001 |
| Hemorrhagic stroke | 2.61 | | 1.899-3.588 | <0.001 |
| Ischemic stroke | 1.533 | | 0.977-2.406 | .063 |
| Epilepsy | 1.537 | | 0.961-2.46 | .073 |
| Pneumonia | 4.57 | | 3.361-6.212 | <0.001 |
| Sepsis | 1.019 | | 0.765-1.356 | .9 |
| Various scores | |  |  |  |
| APSⅢ | 1.016 | | 1.012-1.021 | <0.001 |
| OASIS | 1.033 | | 1.017-1.048 | <0.001 |
| SOFA score | 1.009 | | 0.978-1.042 | .569 |
| GCS score | .82 | | 0.794-0.847 | <0.001 |
| Laboratory tests | |  |  |  |
| RBC count | 1.145 | | 0.989-1.326 | 0.070 |
| WBC count | 1.007 | | 0.994-1.02 | 0.291 |
| PLT count | 1.001 | | 1-1.002 | 0.110 |
| Hb | 1.050 | | 0.999-1.103 | 0.056 |
| Scr | 0.832 | | 0.731-0.948 | 0.006 |
| Vital signs | |  |  |  |
| T | 1.097 | | 0.899-1.338 | 0.362 |
| HR | 0.999 | | 0.991-1.006 | 0.728 |
| SBP | 1.016 | | 1.009-1.023 | 0.000 |
| DBP | 1.000 | | 0.998-1.002 | 0.895 |
| RR | 1.000 | | 0.999-1 | 0.873 |
| SaO_2_ | 1.022 | | 0.96-1.089 | 0.492 |
| Left pupil reaction to light | |  |  |  |
| Brisk | Ref. | | Ref. |  |
| Sluggish | 30.686 | | 22.342-42.146 | <0.001 |
| Non-reactive | 64.322 | | 39.497-104.751 | <0.001 |
| Right pupil reaction to light | |  |  |  |
| Brisk | Ref. | | Ref. |  |
| Sluggish | 25.726 | | 18.787-35.227 | <0.001 |
| Non-reactive | 54.618 | | 33.546-88.926 | <0.001 |
| Blood gas analysis | |  |  |  |
| Ph | 746.458 | | 78.591-7089.828 | <0.001 |
| Lactic acids | 0.937 | | 0.821-1.069 | 0.332 |
| PO_2_ | 1.003 | | 1.001-1.004 | 0.005 |
| PCO_2_ | 0.994 | | 0.98-1.009 | 0.464 |
| IO | 1.001 | | 1-1.002 | 0.261 |
| Treatment received | |  |  |  |
| VAT | 1.436 | | 1.1-1.874 | .008 |
| CRRT | 1.367 | | 0.881-2.122 | 0.163 |
| PN | 2.935 | | 1.982-4.347 | <0.001 |

**Supplementary Table 3.** Baseline characteristics of patients in the early and late tracheotomy groups after PSM.

| Variables | Total  (n=160) | | Early tracheotomy  (n=80) | Late tracheotomy  (n=80) | *p-*value |
| --- | --- | --- | --- | --- | --- |
| Demographic, median (IQR) | |  |  |  |  |
| Age, years | 66.1 (53.8 to 75.3) | | 64.1 (52.2 to 76.2) | 66.8 (57.1 to 74.6) | .858 |
| Sex, male, n(%) | 104 (65%) | | 53 (66.2%) | 51 (63.8%) | .868 |
| BMI, kg/m^2^, | 26.6 (23.8 to 31.3) | | 27.3 (23.8 to 31.3) | 26.2 (23.4 to 31.5) | .400 |
| Comorbidity, n (%) | |  |  |  |  |
| Hypertension | 69 (43.1%) | | 36 (45%) | 33 (41.2%) | .750 |
| Diabetes | 37 (23.1%) | | 21 (26.2%) | 16 (20%) | .453 |
| CAD | 27 (16.9%) | | 13 (16.2%) | 14 (17.5%) | 1.000 |
| CHF | 19 (11.9%) | | 9 (11.2%) | 10 (12.5%) | 1.000 |
| COPD | 4 (2.5%) | | 2 (2.5%) | 2 (2.5%) | 1.000 |
| Traumatic intracerebral hemorrhage | 21 (13.1%) | | 12 (15%) | 9 (11.2%) | .640 |
| Hemorrhagic stroke | 41 (25.6%) | | 20 (25%) | 21 (26.2%) | 1.000 |
| Ischemic stroke | 9 (5.6%) | | 5 (6.2%) | 4 (5%) | 1.000 |
| Epilepsy | 8 (5%) | | 4 (5%) | 4 (5%) | 1.000 |
| Pneumonia | 137 (85.6%) | | 67 (83.8%) | 70 (87.5%) | .652 |
| Sepsis | 25 (15.6%) | | 11 (13.8%) | 14 (17.5%) | .663 |
| Various scores, median (IQR) | |  |  |  |  |
| APSⅢ, mean±SD | 62.0 (51.5 to 79.0) | | 65.0 (51.5 to 82.5) | 62.0 (51.5 to 74.5) | .502 |
| OASIS, mean±SD | 40.0 ± 7.5 | | 39.9 ± 7.9 | 40.2 ± 7.2 | .794 |
| SOFA score | 6.0 (5.0 to 9.0) | | 6.0 (5.0 to 9.0) | 6.0 (4.0 to 9.0) | .944 |
| GCS score | 8.0 (6.0 to 10.0) | | 7.0 (4.0 to 10.0) | 9.0 (6.0 to 10.0) | .328 |
| Laboratory tests, median (IQR) | |  |  |  |  |
| RBC count, ×10^12^/L, mean±SD | 3.9 ± 0.8 | | 3.9 ± 0.7 | 3.9 ± 0.9 | .923 |
| WBC count, ×10^9^/L | 12.3 (9.5 to 17.2) | | 12.6 (9.7 to 17.2) | 11.8 (8.9 to 16.9) | .512 |
| PLT count, ×10^9^/L | 238.0 (177.0 to 295.5) | | 232.0 (180.0 to 305.0) | 240.5 (176.0 to 292.0) | .761 |
| Hb, g/dl, mean±SD | 11.8 ± 2.3 | | 11.9 ± 2.3 | 11.8 ± 2.3 | .858 |
| Scr, mg/dl | 0.9 (0.7 to 1.2) | | 0.9 (0.7 to 1.2) | 0.9 (0.7 to 1.3) | .833 |
| Vital signs, median (IQR) | |  |  |  |  |
| T, ℃ | 37.2 (36.8 to 37.5) | | 37.2 (36.8 to 37.6) | 37.2 (36.8 to 37.4) | .646 |
| HR, n/min | 83.0 (73.7 to 93.7) | | 83.1 (73.5 to 94.8) | 82.8 (74.7 to 92.2) | .866 |
| SBP, mmHg | 119.4 (106.9 to 131.2) | | 119.2 (107.2 to 131.6) | 119.4 (105.6 to 131.1) | .891 |
| DBP, mmHg | 60.6 (53.6 to 72.3) | | 61.1 (54.0 to 72.4) | 60.4 (53.3 to 72.2) | .617 |
| RR, n/min | 19.1 (17.1 to 21.6) | | 19.2 (17.0 to 21.7) | 19.0 (17.4 to 21.6) | .860 |
| SaO_2_, % | 98.7 (96.8 to 99.5) | | 98.8 (96.6 to 99.6) | 98.7 (97.0 to 99.4) | .795 |
| Left pupil reaction to light, n (%) | | | | | .694 |
| Brisk | 56 (35%) | | 26 (32.5%) | 30 (37.5%) |  |
| Sluggish | 68 (42.5%) | | 34 (42.5%) | 34 (42.5%) |  |
| Non-reactive | 36 (22.5%) | | 20 (25%) | 16 (20%) |  |
| Right pupil reaction to light, n (%) | | | | | .767 |
| Brisk | 62 (38.8%) | | 29 (36.2%) | 33 (41.2%) |  |
| Sluggish | 63 (39.4%) | | 32 (40%) | 31 (38.8%) |  |
| Non-reactive | 35 (21.9%) | | 19 (23.8%) | 16 (20%) |  |
| Blood gas analysis, median (IQR) | |  |  |  |  |
| Ph | 7.4 (7.4 to 7.5) | | 7.4 (7.4 to 7.5) | 7.4 (7.4 to 7.5) | .288 |
| Lactic acids, mmol/L | 1.5 (1.0 to 2.5) | | 1.5 (1.0 to 2.4) | 1.4 (1.0 to 2.5) | .875 |
| PO_2,_ mmHg, mean±SD | 113.0 (91.5 to 136.5) | | 109.5 (91.0 to 135.5) | 116.5 (92.5 to 136.5) | .639 |
| PCO_2,_ mmHg | 40.0 (35.0 to 45.5) | | 40.0 (34.5 to 45.5) | 40.0 (35.0 to 46.0) | .838 |
| IO, mmHg, mean±SD | 261.0 (200.0 to 340.0) | | 255.0 (194.0 to 349.0) | 268.8 (204.0 to 337.1) | .793 |
| Treatment received, n (%) | |  |  |  |  |
| VAT | 102 (63.8%) | | 50 (62.5%) | 52 (65%) | .869 |
| CRRT | 3 (1.9%) | | 1 (1.2%) | 2 (2.5%) | 1.000 |
| PN | 3 (1.9%) | | 2 (2.5%) | 1 (1.2%) | 1.000 |

Abbreviations: IQR, interquartile range; SD, standard deviation; BMI, body mass index; CAD, coronary artery disease; CHF, congestive heart failure; COPD, chronic obstructive pulmonary disease; APSⅢ, acute physiology scoreⅢ; OASIS, Oxford Acute Severity of Illness Score; SOFA, sequential organ failure assessment; GCS, glasgow coma scale; RBC, red blood cell; WBC, white blood cell; PLT, platelet; Hb, hemoglobin; SCr, serum creatinine; T, temperature; HR, heart rate; SBP, systolic blood pressure; DBP, diastolic blood pressure; RR, respiratory rate; SaO_2_, arterial oxygen saturation; PO_2_, partial pressure of oxygen; PCO_2_, partial pressure of carbon dioxide; OI, oxygenation index; VAT, vasoactive drug therapy; CRRT, continuous renal replacement therapy; PN, parenteral nutrition.


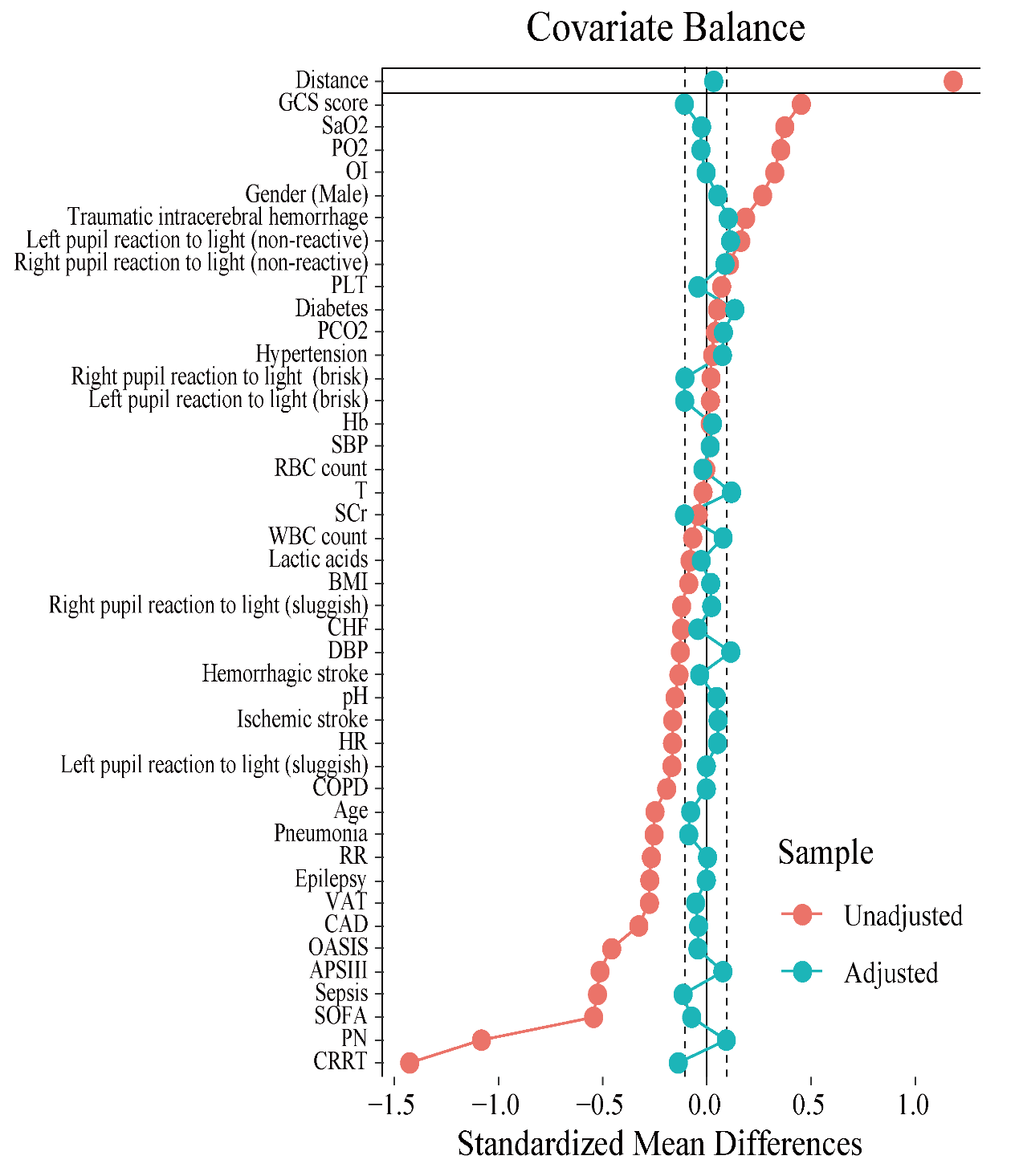


**Supplementary Figure 1**. The SMD values for each variable before and after PSM.
